# Prevention of mortality in acute lung injury induced by oleic acid: Application of polyherbal decoction (bronco T)

**DOI:** 10.3389/fcell.2022.1003767

**Published:** 2022-10-13

**Authors:** Priyanka Mishra, Ratna Pandey, Nikhil Pandey, Suyash Tripathi, Yamini Bhusan Tripathi

**Affiliations:** ^1^ Department of Medicinal Chemistry, Institute of Medical Sciences, Banaras Hindu University, Varanasi, India; ^2^ Department of Physiology, Institute of Medical Sciences, Banaras Hindu University, Varanasi, India; ^3^ Department of Cardiology, Institute of Medical Sciences, Banaras Hindu University, Varanasi, India

**Keywords:** ARDS, ALI, oleic acid, cardiorespiratory, bronco T

## Abstract

Acute lung injury (ALI) is a lethal respiratory disorder; directed uncontrolled inflammation, sloughing of the alveolar cells and their diffusion, and altered cardiorespiratory parameters with a global mortality rate of 40%. This study was designed to assess the preventive effect of a polyherbal decoction (Bronco T, 1.5 g/kg b. w.) on cardiorespiratory variables in oleic acid-induced ALI in rats. Oleic acid increases the level of neutrophil infiltration leading to pulmonary edema and alters the cardiorespiratory dynamics. The adult male rats were surgically cannulated and treated with intravenous oleic acid (0.38 ml/kg b. w.) to establish the ALI model. Bronco T was pre-administered orally 3 hours before oleic acid. The biophysical, histological, biochemical, and molecular effects were compared with dexamethasone (5 mg/kg b. w. i. p.). The animals were randomly divided into control, lethal, standard, and treatment groups. Respiratory frequency (RF), heart rate (HR), and mean arterial pressure (MAP) were recorded on a computerized chart recorder; arterial blood sample was collected to determine PaO_2_/FiO_2_, TNF-α, and MPO. Lipid peroxidation, superoxide dismutase, and catalase activity were evaluated to measure oxidative stress in bronchoalveolar lavage. Additionally, the pulmonary water content, COX-2 expression and histological examination were determined in the lung. A molecular docking study of the active phytoconstituent of BT obtained from HR-LCMS analysis against reported targets (IL-6, COX-2, TNFα, MPO and ENaC) of ALI was carried out. The B.T. pretreatment prevents mortality in comparison to the oleic acid group. It protects the lungs and heart from the detrimental effect of oleic acid, on par with dexamethasone. COX-2 mRNA expression was significantly down-regulated in the treatment group. The reduced level of TNF-α, MPO, SOD and catalase supported the protective effect of B.T. The *in silico* study revealed strong binding interaction between the phytoconstituent (Galangin 3- [galactosyl-(1–4)-rhamnoside and Beta solamarine] of BT and the reported target. The B.T. pre-administration attenuates the oleic acid-induced mortality and cardiorespiratory toxicity.

## Introduction

Acute lung injury is a lethal clinical condition that shuts down the respiratory system in ICU patients. ALI’s advanced clinical stage, known as acute respiratory distress syndrome (ARDS), has a fatality rate of 30–40% (Mikkelsen et al., 2013). A wide range of risk factors, including sepsis, pneumonia and multiple severe traumas can cause ALI (Matthay et al., 2018). The biochemical profile of such patients indicated an elevated level of inflammatory cytokines and reactive oxygen species (Tirunavalli et al., 2021). Although understanding of ALI is improving, its prognosis remains unknown. As a result, it is critical to look for more effective therapy options.

Clinically ALI is characterized by tachypnea, hypotension, and bradycardia, which leads to decreased cardiac output and hypoxemia. Low oxygen saturation combined with tachypnea causes respiratory distress and, eventually, multi-organ failure and death. In ARDS patients, the respiratory rate often increases at first and then gradually declines. As a result, the oxygen level in the pulmonary region is reduced, resulting in severe hypoxemia (Montravers et al., 2015). Additionally, ARDS may be linked to changes in respiratory drive brought on by mechanisms primarily linked to inflammatory lung conditions and altered mechanics. Prior to the disruption of gas exchange, ARDS causes an increase in the respiratory rate in awake, spontaneously breathing rats (Spinelli et al., 2020). Cardiac dysfunction is an important consequence of ARDS that affects mortality and has been attributed to elevated inflammation (Drosatos et al., 2015). In the patient with sepsis mediated ARDS, all blood vessels dilate due to the release of nitric oxide, causing blood pressure to drop resulting in hypotension and bradycardia (Sipmann et al., 2018) (Lee et al., 2019). In a nutshell, when an injury is inflicted on the lungs, within a short duration there is an excessive accumulation of neutrophils along the pulmonary region. A well-established body of literature implicates the increased level of neutrophils with disease severity and mortality rate (Abraham, 2003; Grommes and Soehnlein, 2011). MPO, a prominent neutrophil enzyme, can also reflect the degree of neutrophil infiltration used as a lung injury evaluation index (Bonaventura et al., 2020). Increased neutrophil infiltration along the interstitial lining and alveolar epithelium of the lungs results in the generation of oxidative stress, inflammatory cytokines, and increased fluid accumulation in the lungs (Hattori et al., 2010; Yang et al., 2021). Of which TNFα causes cytotoxicity in fibrotic foci in subpleural areas (Parsons et al., 2005; Oikonomou et al., 2006). Oleic acid upon inoculation is retained by the lungs up to 85 per cent. It induces lung injury with epithelial necrosis and microvascular thrombosis phase, followed by a repair phase with type II cell proliferation and fibrotic foci in subpleural areas. (Derks and Jacobovitz-Derks, 1977).

The experimental model opted here according to the American Thoracic Society (ATS) guidelines (Bernard et al., 1994), where injection of oleic acid (OA) in rats mimics the case of ARDS occurring from multiple traumas that lead to elevated neutrophil levels and oxidative stress. ARDS patients had considerably higher blood levels of OA (Bursten et al., 1996). So, we used this model to justify the protective effect of polyherbal decoction compared to dexamethasone. (Bernard et al., 1994).

Bronco T is a polyherbal decoction which was developed from the Ayurvedic formula “*Shirishadi Kasaya*” (Formula in Table 1). According to Ayurveda, the term “Shirish” means antitoxin (Yadav et al., 2012) and the formulation was developed by late Professor SN Tripathi and his team at the Institute of Medical Sciences BHU for the management of histamine-induced anaphylactic shock in guinea pigs (Tripathi et al., 1983). Corticosteroids such as dexamethasone, triamcinolone acetonide, flunisolide, etc*.* are widely used to treat inflammation such as acute lung injury (Hu et al., 2017). However, their clinical effectiveness is still debatable as their long-term use could lead to Intensive care unit–acquired weakness (ICU- AW), hyperglycemia and osteoporosis (Kramer, 2017; Landolf et al., 2022). Therefore, it is necessary to search for an alternative option with a low risk-to-benefit ratio. Our previous work reported a significant action of Bronco T in improving survival time and cardiopulmonary parameters in LPS-induced septicemia (Mishra et al., 2022). The present work was designed to assess the effect of Bronco T in oleic acid-induced ALI on biophysical, biochemical, histological, and molecular parameters. Bronco T (*Shirishadi Kasaya*) was hypothesized to protect against ALI progression in adult male rats by improving survival time and cardiorespiratory variables.

**TABLE 1:** Composition of bronco T (per 25 gm).

S.No	Plants	Composition
1	Sirisha (*Albizia lebbeck*)	11.25 g w/w
2	Kantkari (*Solanum virginianum*)	5 g w/w
3	Vasaka (*Justicia adhatoda*)	5 g w/w
4	Madhuyasthi (*Glycyrrhiza glabra*)	2.5 g w/w
5	Tejpatra (*Cinnamomum tamala*)	1.25 g w/w

## Materials and methods

### Chemical and reagents

Urethane (U 2500) was obtained from Sigma Aldrich (Missouri, United States) and oleic acid (RM5371) was obtained from Hi-Media Laboratories (Mumbai, India). Dexamethasone sold as Dexona Injection (4 mg per ml-Zydus Cadila, Ahmedabad, India), and Bronco-T (70 g, Batch No-161, Surya Pharmaceuticals, Varanasi, India) were purchased from the local market. TNF-α ELISA kit was obtained from the Elabscience (Texas, United States) rat ELISA kit (E-EL-R2856). The MPO ELISA kit was obtained from the Real gene (Ghaziabad, India) (Cat No. 3100574, Lot No.17517601575). The primers for RT-PCR were obtained from Euro films (Bengaluru, India) and TRIzol, DEPC, and Superscript II RNase H- reverse transcriptase (RT) were obtained from Fermentas, Thermo Fisher Scientific (Waltham, MA, USA). The inbred adult albino rats of the Charles Foster strain (175–225 gm) were acquired from the central animal house of Banaras Hindu University. Cardiorespiratory responses in live rats were recorded using Lab Chart 7 (life sciences data acquisition software) provided by AD instruments (Melbourne, Australia).

### Drug management

Dexamethasone (DEX) was diluted with normal saline as per the dose requirement (5 mg/kg b. w.) for experiments. Bronco T was prepared as per the leaflet information. In brief 15 g of the Bronco T was carefully weighed, crushed and transferred to the conical flask,100 ml of distilled water was added and kept in a water bath to reduce the volume to 25 ml. Then, the decoction was filtered with a strainer and kept at a final concentration of 15 g/25 ml as a stock solution from which the working solution (1.5 g/kg b. w. and 0.75 g/kg b. w.) were prepared. The phytochemical profile of BT was done through HRLC-MS(IIT-Bombay, India).

### Animal treatment

The inbred adult albino rats of the Charles Foster strain (175–225 gm) were acquired from the central animal house of the university. Charles Foster rats are able to mimic the pathological signs of this syndrome to a greater extent than any other model (Schuster, 1994; Matute-Bello et al., 2008); it also provides feasibility in terms of handling and surgical procedure. Only male rats were used to avoid possible effects of gender variance. Animals were housed in standard conditions with a 12-h day/night cycle and allowed to acclimatize for a week. The standard animal diet (Krishna Valley Agrotech Private Ltd., India) and drinking water were provided *ad libitum*.

The ALI rodent model was established by lethal bolus dose of oleic acid (75 µl) which corresponded to 0.38 ml/kg b. w. (Akella et al., 2014; Sharma et al., 2016; Kumar et al., 2021). in group II. OA was injected in all the groups except for group I which was treated with normal saline. In group III, dexamethasone was pre-administered at a dose of 5 mg/kg bw ip (dos Reis et al., 2016) to rats 1 hour before OA administration. In groups, IV and V, the decoction of B.T. was orally given in doses of 1.5 g/kg and 0.75 g/kg body weight 3 h before giving OA injection. (Payani et al., 2019) (Table 2).

**TABLE 2:** Division of groups.

S.No	Groups	Treatment	Route of administration
1	Group I—Control	Normal saline -NS (75 µl)	Intra venous
2	Group II- Lethal	Oleic Acid (0.38 ml/kg b.w. = 75 µl)	Intra venous
3	Group III Standard	Dexamethasone (5 mg/kg bw) + Oleic Acid (0.38 ml/kg b.w = 75 µl)	Intraperitoneal + Intravenous
4	Group IV- Treatment	B.T. (1.5 g/kg b.w.) + Oleic Acid (75 µl)	Per oral + Intravenous
5	Group V- Treatment	B.T. (0.75 g/kg bw) + Oleic Acid (75 µl)	Per oral + Intravenous

Note: oleic acid, Dexamethasone, and B.T., were dissolved in NS.

### Experimental protocol

**FIGURE 1:**
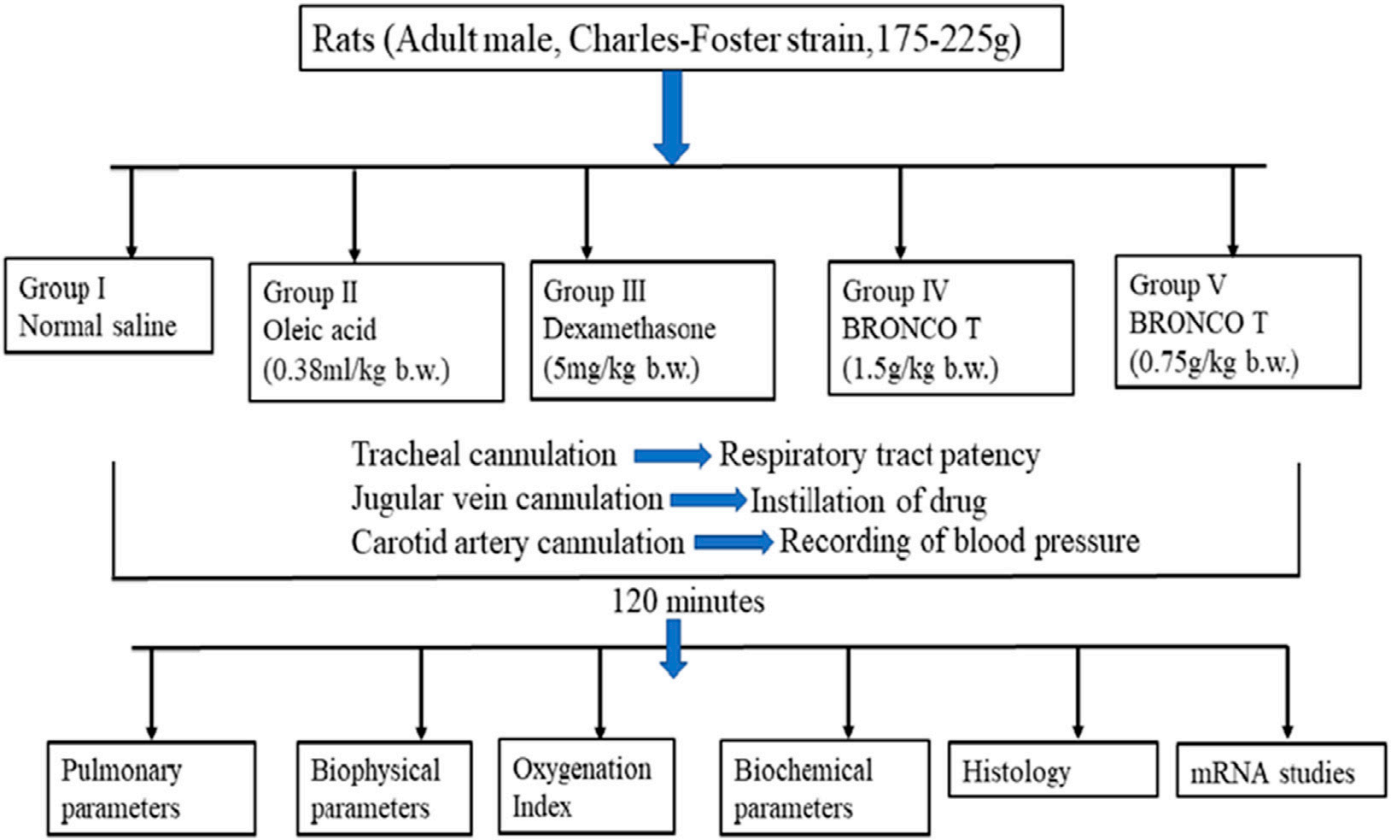
Study design showing the animal group, treatment schedule, and experimental assay.

**FIGURE 2:**
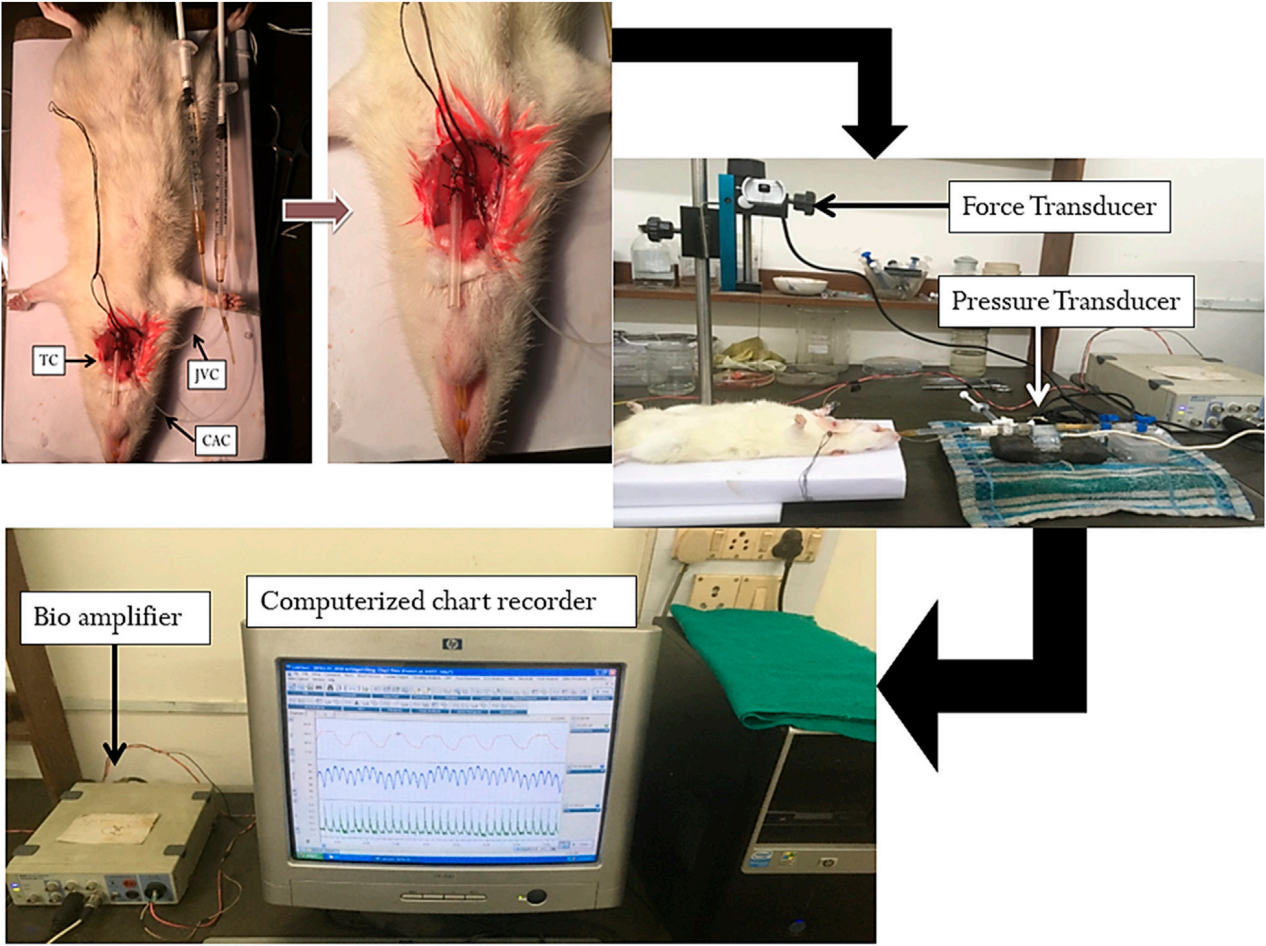
Experimental procedure and setup stating cannulation and recording of biophysical parameters. Experimental procedure and setup that outlines cannulation and recording of biophysical parameters.

The guidelines of the Committee for the Purpose of Control and Supervision of Animal Experiments (CPCSEA) accepted by the Central Animal Ethics Committee of the Banaras Hindu University (letter No. Dean/2021/IAEC/2550) were followed to carry out the experiments in this study (Figure 1). Urethane 1.5 g/kg body weight i. p. was given to anaesthetize the animals. If required, an additional bolus dose of urethane (0.1–0.15 g/kg ip) was injected. The trachea, jugular vein and carotid artery were cannulated to maintain respiration and for drug administration, and to record blood pressure, respectively (Figure 2). The animals were stabilized for 30 min after dissection and cannulation. The animals were randomly divided into five groups control (normal saline), lethal (oleic acid), DEX + OA, BT (1.5 g/kg bw) + OA, BT (0.75 g/kg bw + OA) (n = 6/each group) as described below.

During the experiment for 120 min, all physiological parameters were recorded and before the sacrifice of an animal, bronchoalveolar lavage was performed using ice-cold PBS (Karagiorga et al., 2006) for biochemical estimation and blood was collected, from the right internal carotid artery, for blood gas analysis. Finally, the lungs were excised and one lung lobe was kept to estimate the pulmonary water content, and other lobes were fixed in formaldehyde for histological examination.

### Phytochemical profiling of bronco T and molecular docking

The HR-LCMS analysis of Bronco T was performed at IIT-Bombay using Agilent 6550 iFunnel QTOF LC-MS, Agilent Technologies 1,290 Infinity binary pump, 108 vial autosampler (Agilent Technologies, USA). The detection was done by the Agilent Jet Stream Electron Spray Ionization (AJS-ESI) mode. The phytoconstituents with maximum retention time were subjected to *in silico* molecular docking analysis with the help of YASARA (Krieger & Vriend, 2014) and Discovery studio 2021 (Shree et al., 2022) against reported targets including interleukin-6 (PDB ID: 1ALU) (Li et al., 2008), tumour necrosis factor-alpha (PDB ID: 2AZ5) (Li et al., 2020), cyclo oxygenase-2(PDB ID: 5JW1) (Boyle et al., 2018), myeloperoxidase (PDB ID: 5FIW) (Bonaventura et al., 2020) and epithelial sodium channel (PDB ID: 6BQN) (Gwoździńska et al., 2017).

### Biophysical estimates

#### Determination of survival time

Mortality was characterized by a flat line response in cardiorespiratory responses (RR, MAP, and HR) as observed from the laboratory chart recorder (AD Instrument, Melbourne, Australia).

#### Determination of respiratory rate

It was recorded by securing the skin over the xiphisternum of the rat in a supine position with help of a cotton suture thread and half circle suture needle. With the help of artery forceps and tissue forceps the needle was carefully inserted over the skin, tied and the other end of the thread was connected to the Lab Chart recorder (AD Instrument, Australia) through a needle attached with the force-displacement transducer. The deflection of thread corresponded to the respiratory movement of the rat which was recorded.

#### Determination of mean arterial pressure

At the beginning of each experiment, the pressure transducer was calibrated and the carotid artery was connected to it through a three-way stop clock. Blood pressure was recorded by connecting the carotid artery cannula to the pressure transducer to a Lab Chart recorder (AD Instrument, Melbourne, Australia) through a bridge amplifier. MAP was determined by the formula:

$$MAP=Diastolic\;\mathrm{Pr}essure+\frac{1}{3}\left(Pulse\;\mathrm{Pr}essure\right),$$

where Pulse Pr⁡essure=Systolic pressure−Diastolic Pr⁡essure

#### Determination of heart rate

It was manually calculated from the R-R interval by recording the electrocardiograph by connecting the needle electrode to the chart recorder through a bio amplifier using standard limb lead II configuration in the rats.

### Determination of oxygenation index

#### SpO_2_ and PaO_2_/FiO_2_ ratio

SpO_2_ was obtained using a pulse oximeter (AD Instrument). Its one end was connected to the tail vein of the rat and another was connected to the chart recorder through a bio amplifier and readings were recorded within 15 min of saline administration in the control group and 15 min after oleic acid administration in other groups. PaO_2_/FiO_2_ ratio was estimated by an ABG analyzer (Roche OMNI gas analyser) by collecting a 100 µl blood sample from the right internal carotid artery using a heparinized syringe (5000 IU/ml, 2% in normal saline).

### Determination of changes in lung morphology and histology

#### Determination of the pulmonary water content

It was determined by the physical method as described earlier ((Kaner et al., 2012; Kristof et al., 2012)Pandey et al., 2017). At the end of each experiment, the lungs were excised. One lung was dried on tissue paper to remove the external moisture and weighed. After that, it was dried in an electric oven (at 65°C for 48 h) to a constant dry weight. The difference between wet weight and dry weight was reported as the water content in the lung and reported in terms of the percentage of wet weight. It was calculated by the following formula:

$$pulmonary\;water\;content=\left[\frac{Wet\;Lung\;weight-Dry\;lung\;weight}{Wet\;lung\;weight}\right]*100$$

#### Histology of lungs

Another excised lung was used for this study. The surface morphology was observed and photographed. Then it was washed with ice cold phosphate buffer saline and preserved in 10% formalin solution for 24 h. Further, it was subjected to standard histological protocol and stained with hematoxylin (H) and eosin (E) for microscopic examination (×10, ×40 magnifications).

#### Lung injury score

The lung injury scoring system was adapted from the Matute-Bello Murray Scoring system (Fan et al., 2018) (Fang et al., 2018) for semiquantitative measurements in five eye fields and reported in the following parameters (Table 3).

**TABLE 3:** Lung injury scoring system.

S.No.	Parameters	0	Grade-1	Grade-2
A	Neutrophils in Alveolar space	None	1–5	>5
B	Neutrophils in Interstitial space	None	1–5	>5
C	Presence of Hyaline Membranes	None	1	>1
D	Number of proteinaceous debris	None	1	>1
E	The extent of alveolar septal thickening	<2x	2x-4x	>4x

Score=[20*(A)+14*(B)+7*(D)+2(E)]/(number of fields * 100) .

### Determination of the level of myeloperoxidase, malondialdehyde, and TNFα level

The level of myeloperoxidase (MPO) was estimated in lung homogenate in freshly prepared lysis buffer, which was centrifuged for 5 min at 5000 RPM. The supernatant was collected and immediately estimated using the procedure stated by the Real gene rat MPO ELISA kit (Cat No. 3100574, Lot No.17517601575) at 435 nm (Klebanoff, 2005). The level of lipid peroxides was measured as a a thiobarbituric acid reacting substance (TBARS) and expressed as equivalent to malondialdehyde (MDA) using 1,1,3,3-tetra methoxy propane (TEP) as standard in lung homogenate (Ohkawa et al., 1979). It was expressed in terms of μ mol/mg protein. The presence of reactive substances with thiobarbituric acid (TBARS) was detected by monitoring the changes in absorbance using a microplate reader at 535 nm. TNF-α was estimated in the lung homogenate through a procedure stated by the Elabscience rat ELISA kit (E-EL-R2856).

### Determination of oxidative stress parameter

Bronchoalveolar lavage fluid (BALF) was acquired from the left upper lung as previously described (Cho et al., 2020). Superoxide dismutase (SOD) activity was measured in terms of inhibition of reduction of nitro blue tetrazolium (NBT) in the presence of riboflavin, as described (Beauchamp and Fridovich, 1971) with slight modification (Nagwani and Tripathi, 2010). Catalase enzyme activity was measured using the Aebi method by monitoring H_2_O_2_ breakdown at 240 nm (Aebi, 1984).

### Expression of mRNA level of COX-2 in lung homogenate

The total RNA was extracted from the lungs of the rat using TRIzol reagent. According to its user manual, RNA dissolved in water treated with diethyl pyrocarbonate (DEPC) was quantified by using the spectrophotometer. 5 mg of total RNA was reverse transcribed with Superscript II RNase H- reverse transcriptase (RT) using random hexamers according to the manufacturer’s instructions (Fermentas, Thermo Fisher Scientific, Waltham, MA, USA). Synthesized cDNA was stored at - 20°C to be directly used for (Reverse Transcriptase Polymerase Chain Reaction) RTPCR reactions. Specific oligonucleotides were used for the analysis of COX-2 (forward (F) 5’ - AAA​GGC​CTC​CAT​TGA​CCA​GA -3′, Reverse(R) 5′- GTG​CTC​GGC​TTC​CAG​TAT​TG-3′), product size 373 bp and GAPDH (glyceraldehyde 3- phosphate dehydrogenase) 5′- AGT​GAG​GAG​CAG​GTT​GAG​GA-3′ (Forward primer) and 5′-GAG​GAG​GGG​AGA​TGA​TGT​GA-3’ (Reverse primer), product size 244 bp were synthesized from Euro film, India. The PCR reactions were performed in 25 µl reaction mixtures containing 2 µl cDNA, 2.0 mM of dNTP, 2.5 µl of 10 x standard Taq reaction buffer, and 1.0 unit of Taq DNA polymerase (New England BioLabs, Inc.) and 5 μmol of each primer. The reactions were carried out in Thermo Fischer MiniAmp thermal cycler. PCR steps for COX-2: initial denaturation at 94°C for 5 min- 1 cycle, followed by 35 cycles of 94 C for 45 s, 55 C for 45 s, 72 C for 1 min and final extension at 72°C for 10 min. The steps for GAPDH: initial denaturation at 94°C for 3 min, followed by 35 cycles of 94°C for 30 s, 55°C for 30 s, 72°C for 1 min and final extension at 72°C for 8 min. The amplified products were separated on 1.5% agarose gel electrophoresis containing ethidium bromide. The intensity of COX-2 was determined using image quant Omega FlourTM (GEL Company, USA) using Omega Fluor Acquisition software. The expression of COX-2 was expressed as per cent band intensity relative to that of GAPDH. All RT-PCR experiments were performed in triplicate with the same results, and the best result was provided in the results section.

### Data analysis of data

The changes in RR, HR and MAP were expressed as % of the initial (0 min). The data were pooled and the mean ± standard deviation (SD) was calculated (n = 6). Graph pad prism 8.0 was used for statistical and graphical analysis. Comparisons between groups were performed using repeated measure one-way ANOVA (Tukey’s multiple comparison tests) to compare the mean values of each group with each other. Statistical analysis was carried out using a one-sample T-test and Wilcoxon test for cardiorespiratory parameters. Kaplan-Meier survival curves and logarithmic rank test (Mantel-Haenszel) were used to compare survival time. *p < 0.05, ***p < 0.001, and ****p < 0.0001 is significantly different from the normal group; ^*#*^ *p < 0.05* ###*p < 0.001* and ^*####*^ *p < 0.0001* are significantly different from the lethal group.

## Results

### Phytochemical profiling of bronco T

**FIGURE 3:**
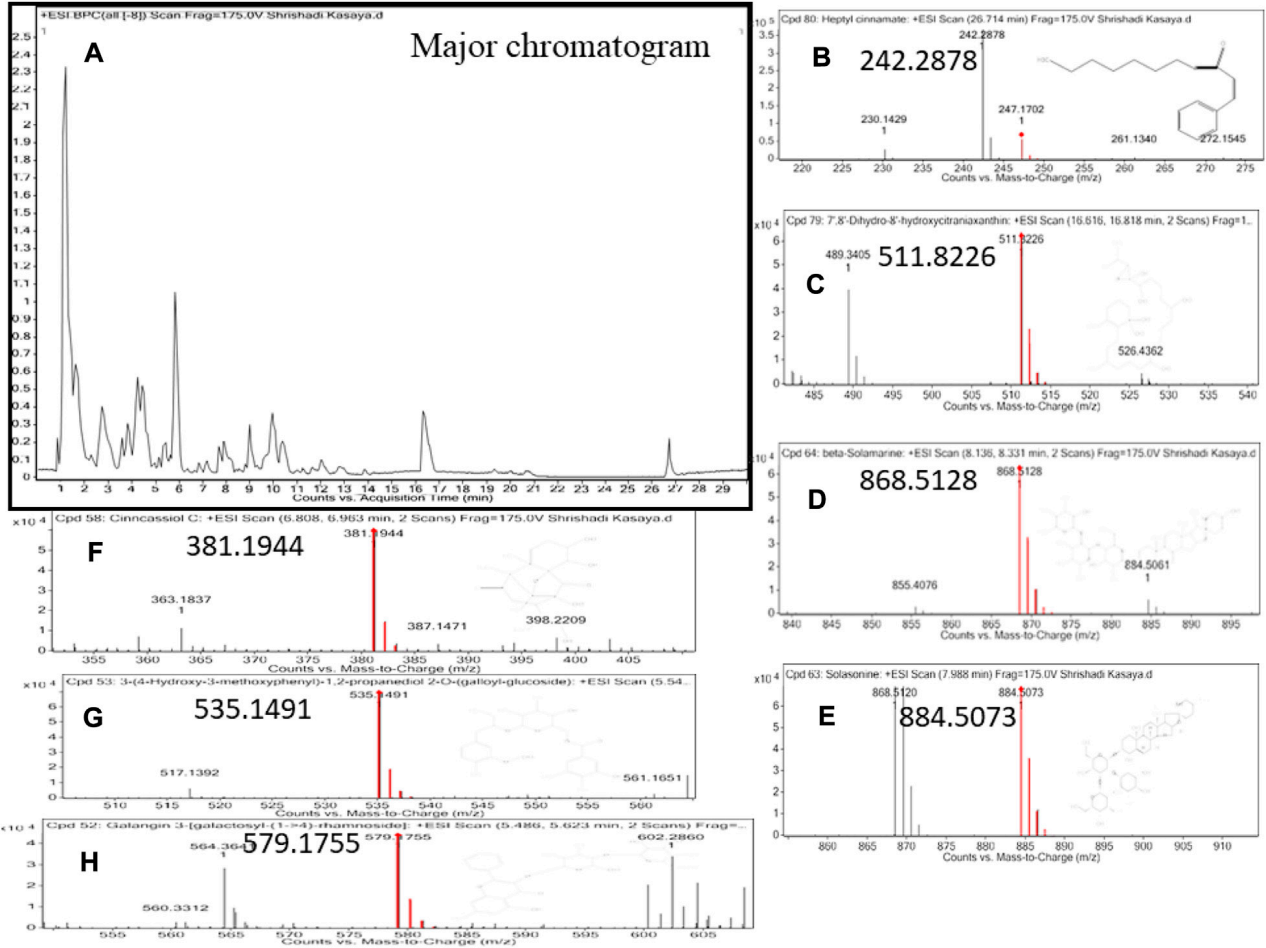
High-resolution liquid chromatography-mass spectroscopy of Bronco T showing the Major chromatogram. High-resolution liquid chromatography-mass spectroscopy of Bronco T showing the Major chromatogram **(A)** and the identified phytoconstituent with the highest retention time, which is active against respiratory disorders **(B)**: heptyl cinnamate, **(C)** 7′, 8′-dihydro-8′-hydroxycitraniaxanthin, **(D)** beta-solamarine, **(E)** Solasonine, **(F)** Cinncassiol C, **(G)** 3- (4-hydroxy-3-methoxyphenyl) -1,2-propanediol 2-O- (gallolyl-glucoside), **(H)** Galangin 3- [galactosyl-(1->4)-rhamnoside]).

**FIGURE 4:**
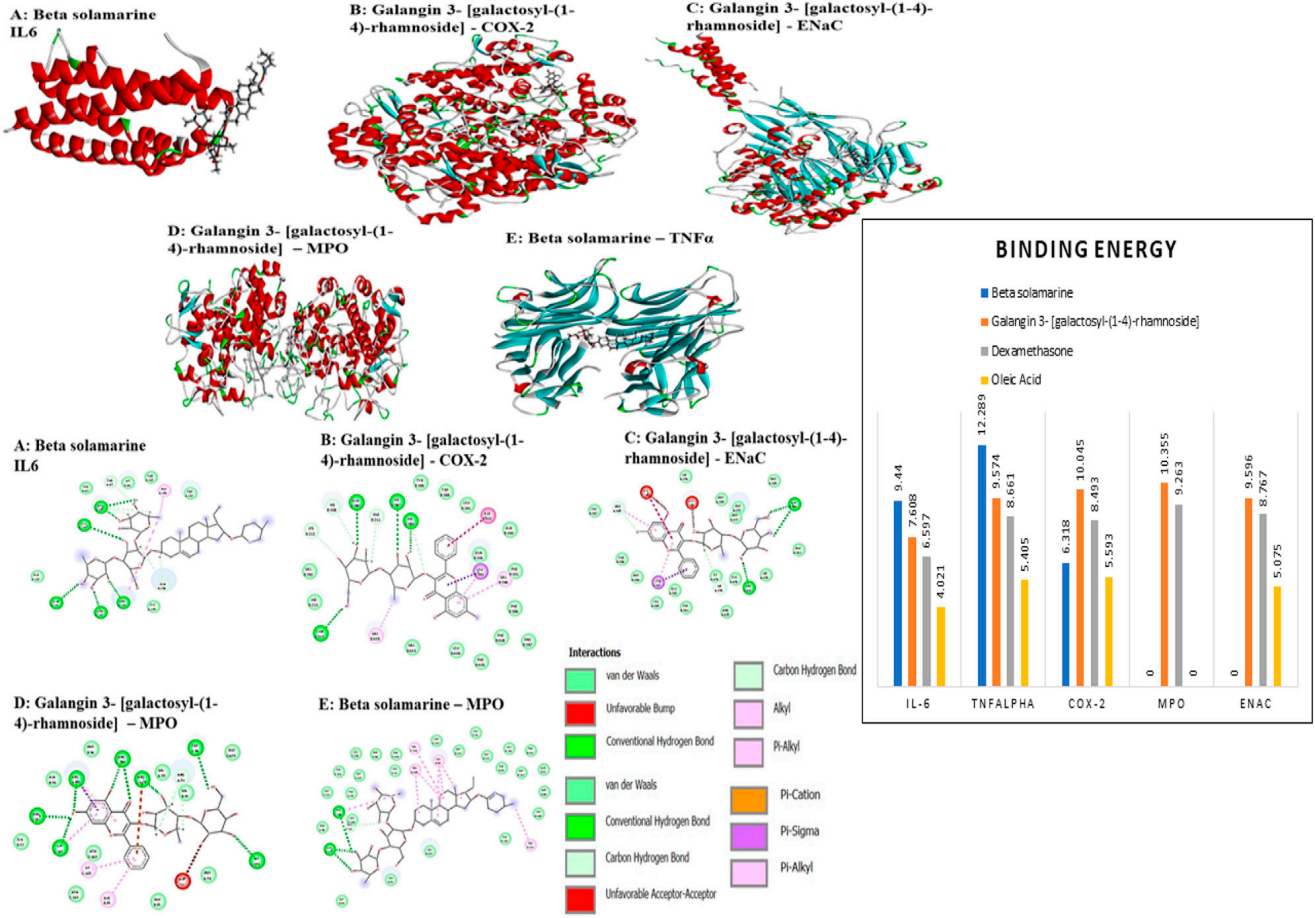
Docking interaction and binding energy comparison between phytoconstituent and molecular targets of ALI. Molecular coupling analysis revealed two phytoconstituents, i.e., beta-Solamarine and Galangin-3- [galactosyl-(1–4)-rhamnoside showed significant binding energy to the reported targets compared to dexamethasone. A pictorial representation of the 2D receptor-ligand interaction **(A)**: Beta-solamarine with IL-6, **(B)** Galangin-3- [galactosyl-(1–4)-rhamnoside with COX-2, **(C)** Galangin-3- [galactosyl-(1–4)-rhamnoside with ENaC, **(D)** Galangin-3- [galactosyl-(1–4)-rhamnoside with MPO and **(E)** Beta solamarine with MPO) and a comparison of binding energy in the graph are shown above.

A total of 77 Bronco T phytocompounds were reported as shown in the additional file with their reported activity against respiratory disorders. The major constituent present with maximum retention time were heptyl cinnamate (26.728 min), 7′,8′-Dihydro-8′- hydroxycitraniaxanthin (16.744 min), beta-Solamarine (8.249 min), Solasonine (8.002), Cinncassiol C (6.9 min), 3-(4- Hydroxy-3- methoxyphenyl)-1,2- propanediol 2-O-(galloyl-glucoside) (5.659min) and Galangin 3- [galactosyl-(1->4)-rhamnoside] (5.573 min). The major chromatogram and spectra of major compounds are shown in Figures 3A–H. Among these beta-Solamarine and Galangin 3- [galactosyl-(1->4)-rhamnoside] showed the highest binding affinity against the reported targets of ALI in comparison to dexamethasone as shown in Figure 4.

### Effect on biophysical parameters

#### Effect on survival time

**FIGURE 5:**
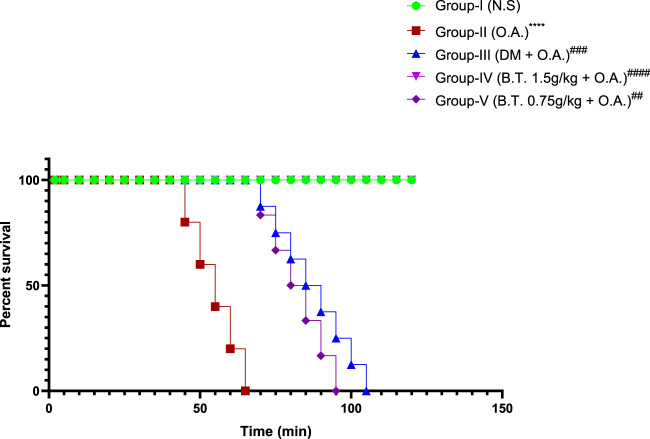
Kaplan-Meier survival plot. Kaplan-Meier survival plot. Overall survival in group IV (B.T. 1.5 g/kg b. w.) was similar to group I (normal saline) and significantly different from group II (oleic acid) as per the log-rank (Mantel–Haenszel) test.*, p < 0.05, ***, p < 0.001,****, p < 0.0001 in comparison to group I and ^*#*^ *,p < 0.05,* ^*###*^ *,p < 0.001 and* ^*####*^ *,p < 0.0001* in comparison to group II.

**FIGURE 6:**
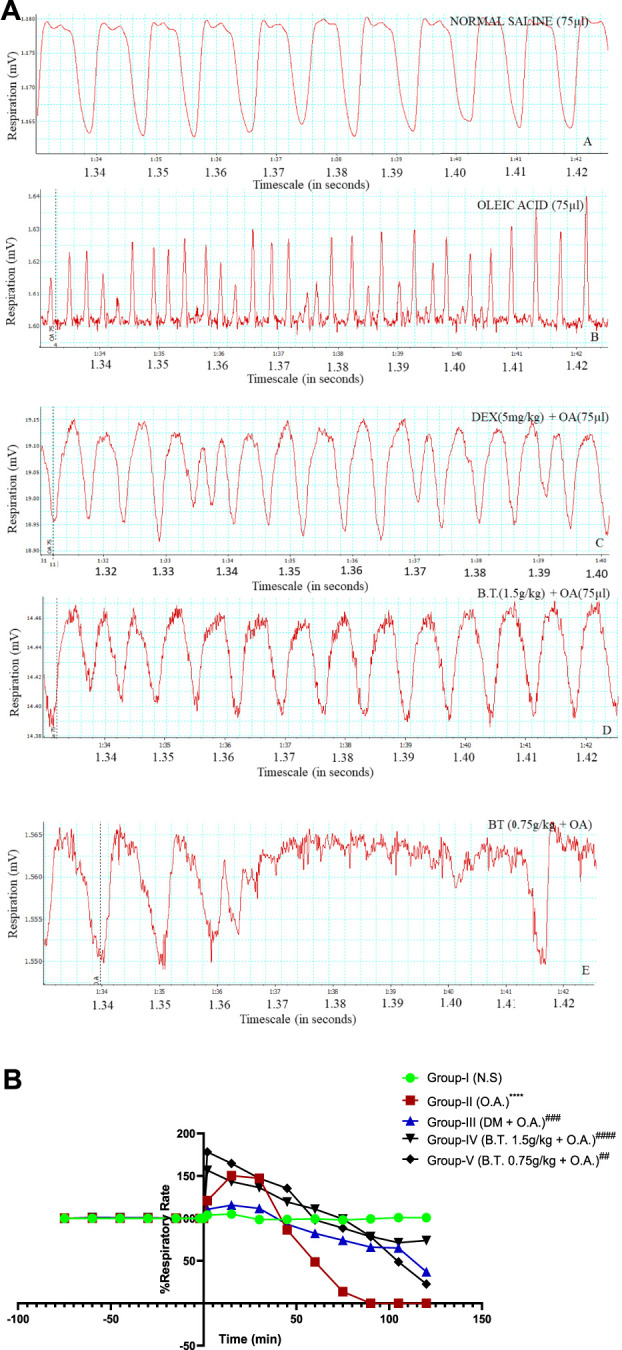
**(A,B)**: Respiration rate comparison between the groups through chart recorder **(A)**and statistically **(B)**. **(A)**: Respiration rate frequency denoted by red colour in real-time in groups I (A), II (B), III (C), IV (D) and V (E) at a 10:1 aspect ratio obtained from a computerized chart recorder where *x*-axis represents Respiration (mV) and *y*-axis represent timeline. Group I shows the basal value of RR, whereas tachypnea is prominent in group II. This was rectified in treatment groups III and IV as near normal frequency can be seen after instillation of oleic acid (denoted by a dotted line) instillation. **(B)**: Respiration rate calculated as percentage initial at 0 min (denotes the time of oleic acid instillation except for group I). Results are expressed as mean ± SD (n = 6). Statistical analysis was carried out using one-sample T-test and Wilcoxon test where *, p < 0.05, ***, p < 0.001,****, p < 0.0001 in comparison to group I and ^*#*^ *,p < 0.05,* ^*###*^ *,p < 0.001 and* ^*####*^ *,p < 0.0001* in comparison to group II.

**FIGURE 7:**
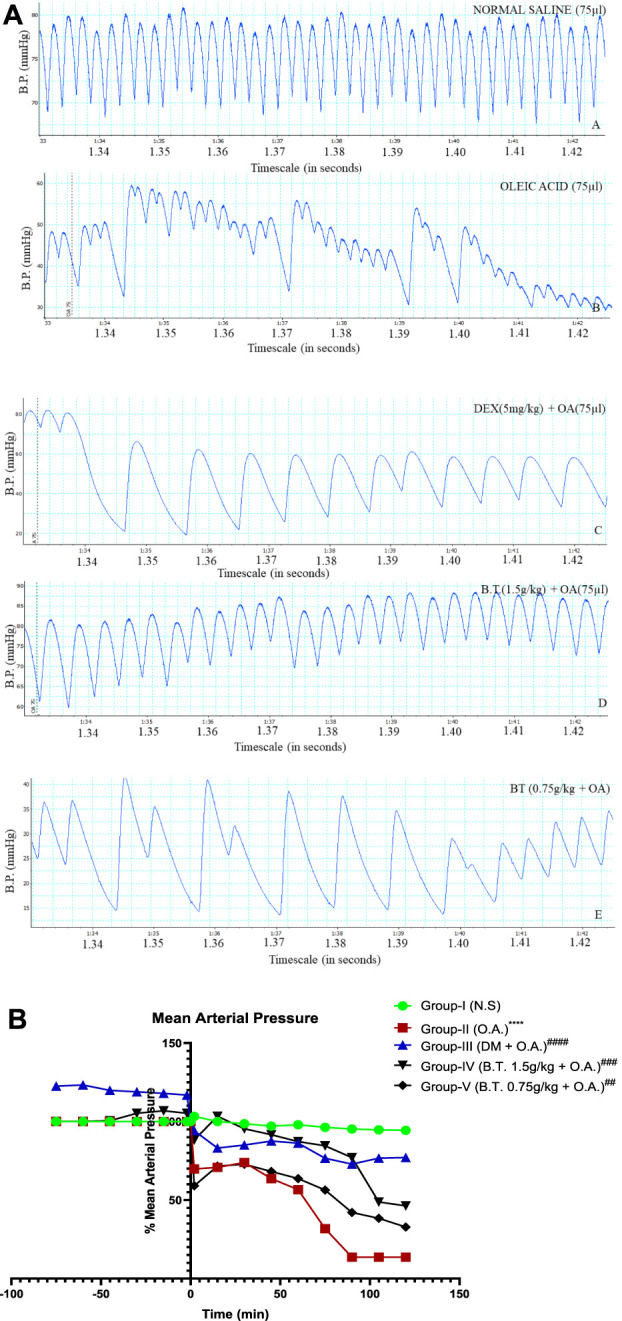
**(A,B)**: Comparison of mean arterial pressure between the groups using the chart recorder **(A)** and statistically **(B)**. **(A)**: Blood pressure recording denoted by blue color in real time in group I, II, III, IV and V at a 10:1 aspect ratio obtained from a computerized chart recorder where *x*-axis represents Blood pressure (mmHg) and *y*-axis represent timeline. Group I shows the basal value of BP (crest denoting systolic and the trough denotes diastolic), while a decrease in BP is prominent in group II. This was rectified in treatment groups III and IV as near normal frequency can be seen after instillation of oleic acid (denoted by a dotted line) instillation. **(B)**: Mean arterial pressure calculated from blood pressure as a percentage of initial at 0 min (denotes the time of instillation of oleic acid, except for group I). Results are expressed as mean ± SD (n = 6). Statistical analysis was carried out using one-sample T-test and Wilcoxon test where *, p < 0.05, ***, p < 0.001, ****, p < 0.0001 in comparison to group I and ^*#*^ *,p < 0.05,* ^*###,*^ *p < 0.001 and* ^####^,*p < 0.0001* in comparison to group II.

**FIGURE 8:**
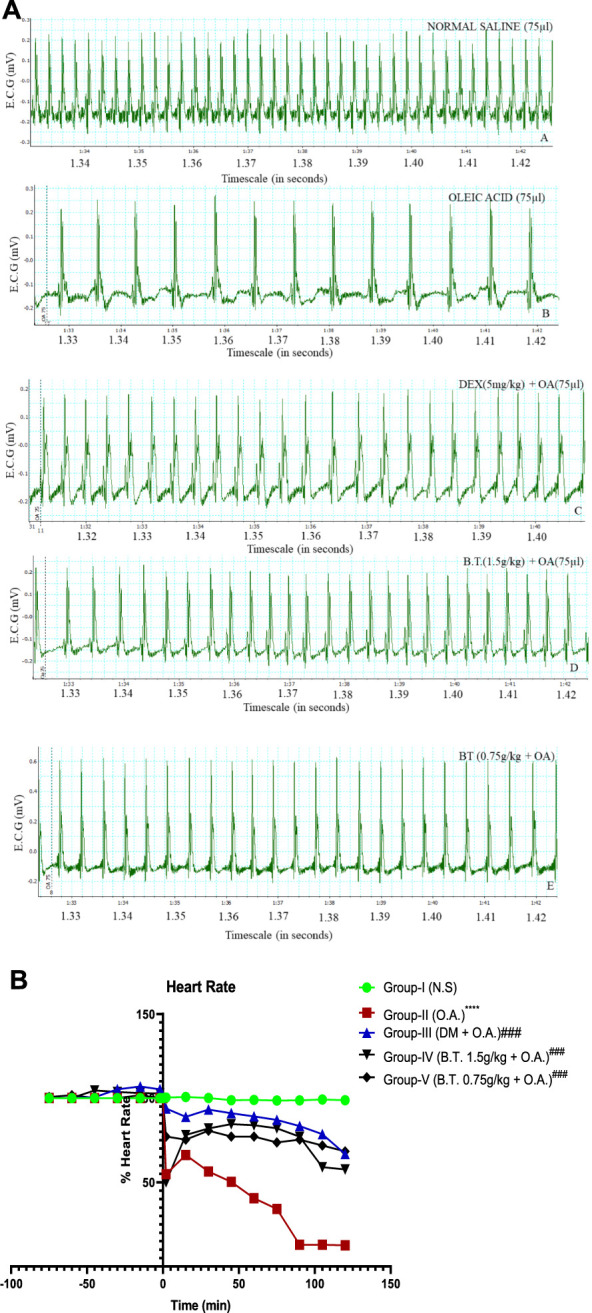
**(A,B)**: Heart rate comparison between the groups through chart recorder **(A)** and statistically **(B)**. **(A)**: Heart rate frequency denoted by green colour in real-time in the groups I, II, III, IV and V at a 10:1 aspect ratio obtained from a computerized chart recorder where *x*-axis represents ECG (mV) and *y*-axis represent timeline. Group I shows the basal HR value, while bradycardia is prominent in group II. This was corrected in treatment group IV, as a near normal frequency can be seen after instillation of oleic acid (denoted as dotted line) instillation. **(B)**: Heart rate calculated as the initial percentage at 0 min (denotes the time of instillation of oleic acid, except for group I). The results are expressed as mean ± SD (*n* = 6). Statistical analysis was performed using the one-sample T test and Wilcoxon test where *, p < 0.05, ***, p < 0.001,****, p < 0.0001 in comparison to group I and ^*#*^ *,p < 0.05,* ^*###*^ *,p < 0.001 and* ^*####*^ *,p < 0.0001* in comparison to group II.

All the animals in group-I (N.S. 75 μl n = 6) survived throughout the experimental period (120 min). In group-II (O.A. 75 μl n = 6), the survival time was reduced to 65.5 min ±3.15, but in treated group III (D 5 mg/kg n = 6), survival time was 110 min ±0.5, significantly higher than in group II. In the Bronco T treated group, a dose-dependent enhancement in survival time was recorded. It was 120 ± 0.2 min for group-IV (B.T. 1.5 g/kg n = 6), and 112 min ±0.2 for group-V (B.T. 0.75 g/kg n = 6) Figure 5. On statistical analysis, the BT dose of 0.75 gm/kg was equivalent to the dose of 5 mg/kg of Dexamethasone (*Chi-square = 109.7, df= 4, p-value < 0.0001,* ^****^) Though, the dose of BT at 1.5 g/kg showed a better survival rate, than Dexamethasone. There was a significant improvement in cardiorespiratory parameters including RR (Figure 6A), MAP (Figure 7A) and HR (Figure 8A), showing a similar pattern to survival time. The response has been presented as a percentage (%) change from the initial response at 0 min, that is, the time of administration of saline in RR (Figure 6B), MAP (Figure 7B) and HR ().

#### Respiratory rate

In group (I), there was no change in RR throughout the observation period. In group II, there was an immediate increase in respiratory rate of about 44% at 2 min after O.A. injection and kept on increasing and became 2.11 times higher than the initial value, at 30 min. However, after this time, there was a progressive drop in respiratory frequency that finally stopped at 75 min with only 1 beat/5 min, leading to death. There were episodes of labored breathing after 30 min between tachypnea episodes which may reduce the level of PaCO_2_ leading to respiratory distress. In the case of group III, the initial response after oleic acid injection was not as pronounced as seen in the group treated with oleic acid, but a gradual decrease in RR was observed with time. In group-IV (B.T. 1.5 g/kg) pre-treated group, there was an initial increase (37.5%) in respiratory rate 2 min after oleic acid injection, but after 15–30 min it returned to normal and the RR was maintained at its level for the entire observation period. However, in group V (B.T. 0.75 g/kg) the tachypnea pattern was similar to the oleic acid treated group (*p < 0.0001,* ^****^). Figures 6A,B

#### Mean arterial pressure

There was no significant change in MAP in group I (3 B) throughout the recording time. In group II, an immediate (2 min) fall (about 45%) in MAP, followed by a recovery pattern of up to 23% by 30 min; and then progressive fall until death (90 min) reaching 0 mm of Hg (*p < 0.0001,* ^****^). However, in group III (D 5 mg/kg) and IV (BT 1.5 g/kg) significantly prevented this fall, suggesting a cardioprotective role. Figures 7A,B

#### Heart rate

In group-I (3C), there was no significant change throughout the experimental period (98.2% ± 0.3). In group II (4C), after OA (75 µl), HR was reduced to 42.45% ± 0.5 and after 90 min the heartbeat stopped. Interestingly, in group III (5C), HR was reduced only by 10% ± 0.9 from group I, which was significantly higher than in group II. However, after 15 min, it was significantly lower than in group I, suggesting that this dose of dexamethasone could not bring the rats to their normal condition. Similarly, in group-IV & V, the BT pretreatment showed a dose-dependent recovery from the oleic acid-induced reduction in heart rate. At a dose of 1.5 g/kg of B.T. (6C), there were 21.7% ± 1.9 reductions after 15 min of OA injection, but no mortality was reported at this dose whereas the rats died on the other tested dose at 112 min (*p < 0.0001,* ^****^). Figures 8A,B

### Oxygenation index

#### PaO_2_/FiO_2_ ratio

**FIGURE 9:**
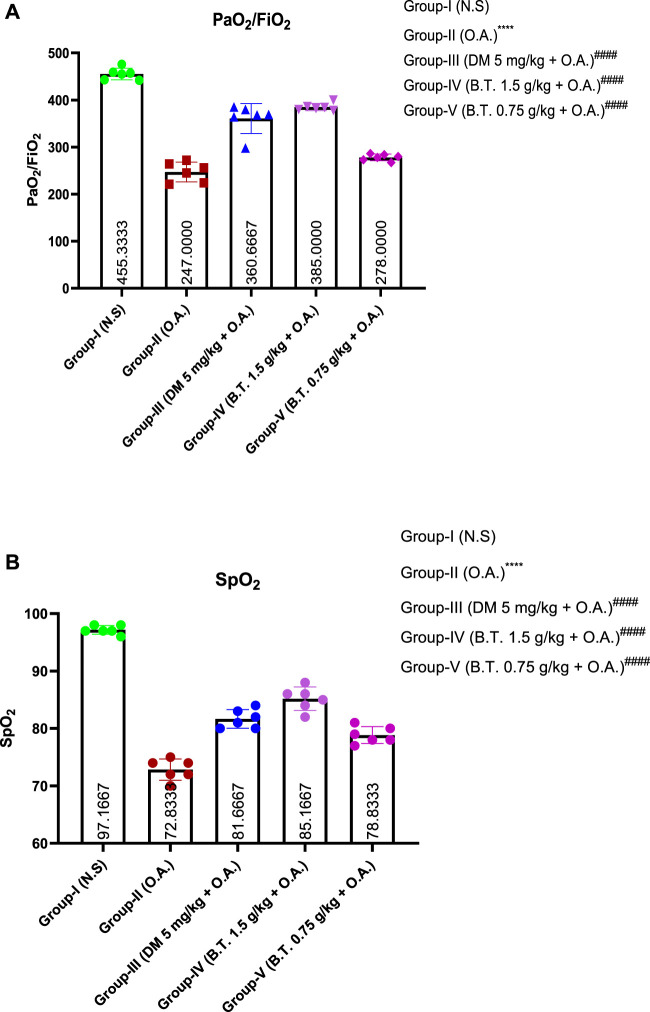
Effect of B.T (1.5 g/kg b. w.) on PaO_2_/FiO_2_ **(A)** and SpO_2_ **(B)** and) in live rats. Effect of B.T. (1.5 g/kg B.W.) on oxygenation index including PaO_2_/FiO_2_ **(A)** and SpO_2_ **(B)** in live rats. A substantial reduction was reported after oleic acid injection in group II in comparison to group I, which was restored back in both group III and group IV. Comparisons between groups were performed using one-way ANOVA (Turkey’s multiple comparison tests) to compare the mean values of each group with each other. (*p < 0.05, ***p < 0.01, and ****p < 0.001 is significantly different from group I; ^*#*^ *,p < 0.05,* ^*###*^ *,p < 0.001 and* ^*####*^ *,p < 0.0001* are significantly different from group II).

The mean ± SD of PaO_2_/FiO_2_ was found in the oleic acid-treated group II was 246 ± 1.8, this value was significantly higher in the BT treated group (1.5 g/kg) (382 ± 2.4), although it was lower than the normal control group (459.3 ± 2.3). The dexamethasone treatment also showed similar, significant improvement (379 ± 2.3) as compared to OA treated group (*F*_*(4, 25)*_ *= 1,483, p < 0.0001,* ^****^). Figure 9A.

#### SpO_2_ level

The mean ± SD of the control group was 97 ± 0.34, whereas it dropped down to 74.2 ± 0.95 in the oleic acid-treated group, but significantly improved in the group of dexamethasone (82.7 ± 0.80) and BT (1.5 gm/kg) treatment (86 ± 1.15) (*F*_*(4, 25)*_ *=147.2, p < 0.0001,* ^****^). Figure 9B.

### Effect on pulmonary water content, lung morphology, and histology

#### Pulmonary water content

**FIGURE 10:**
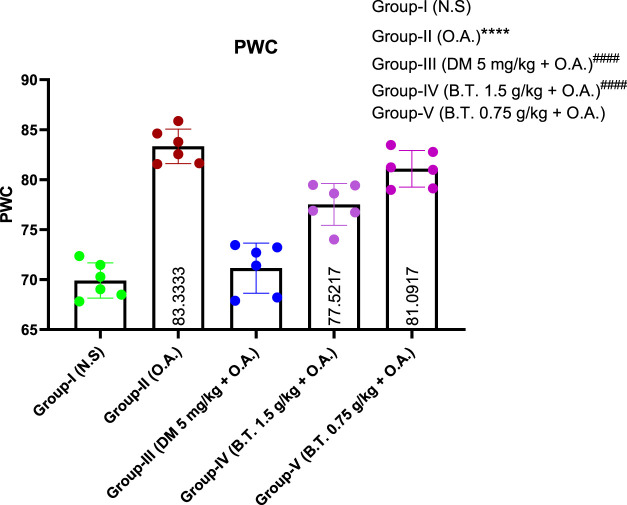
Effect of B.T. (1.5 g/kg B.W.) on the pulmonary water content. Effect of B.T. (1.5 g/kg B.W.) on the pulmonary water content. Group II reported an elevated level of pulmonary oedema which was rectified in the treatment groups III and IV. Comparisons between groups were performed using one-way ANOVA (Turkey’s multiple comparison tests) to compare the mean values of each group with each other. (^*^ *p < 0.05,* ***p < 0.01, and ****p < 0.001 is significantly different from group I; ^*###*^ *p < 0.01 and* ####*p < 0.001* are significantly different from group II).

The individual values of the pulmonary water content of the oleic acid treated group (II) were (83.20 ± 0.61) indicating significant edema, compared to the control group (I) (70.92 ± 1.40). This rise in the water content of the lung due to OA administration was significantly lower in the dexamethasone group (III) (71.95 ± 3.15) and BT (1.5 g/kg b. w.) (73.47 ± 1.04), very close to the normal value. Interestingly, the lowest dose of BT tested (0.75 g/kg bw) did not show any protection and the value was close to the group treated with oleic acid (81.31 ± 1.54) (*F*_*(4, 25)*_ *=134.5, p < 0.0001,* ^****^). Figure 10.

#### Pathological changes of lung tissue in each group

**FIGURE 11:**
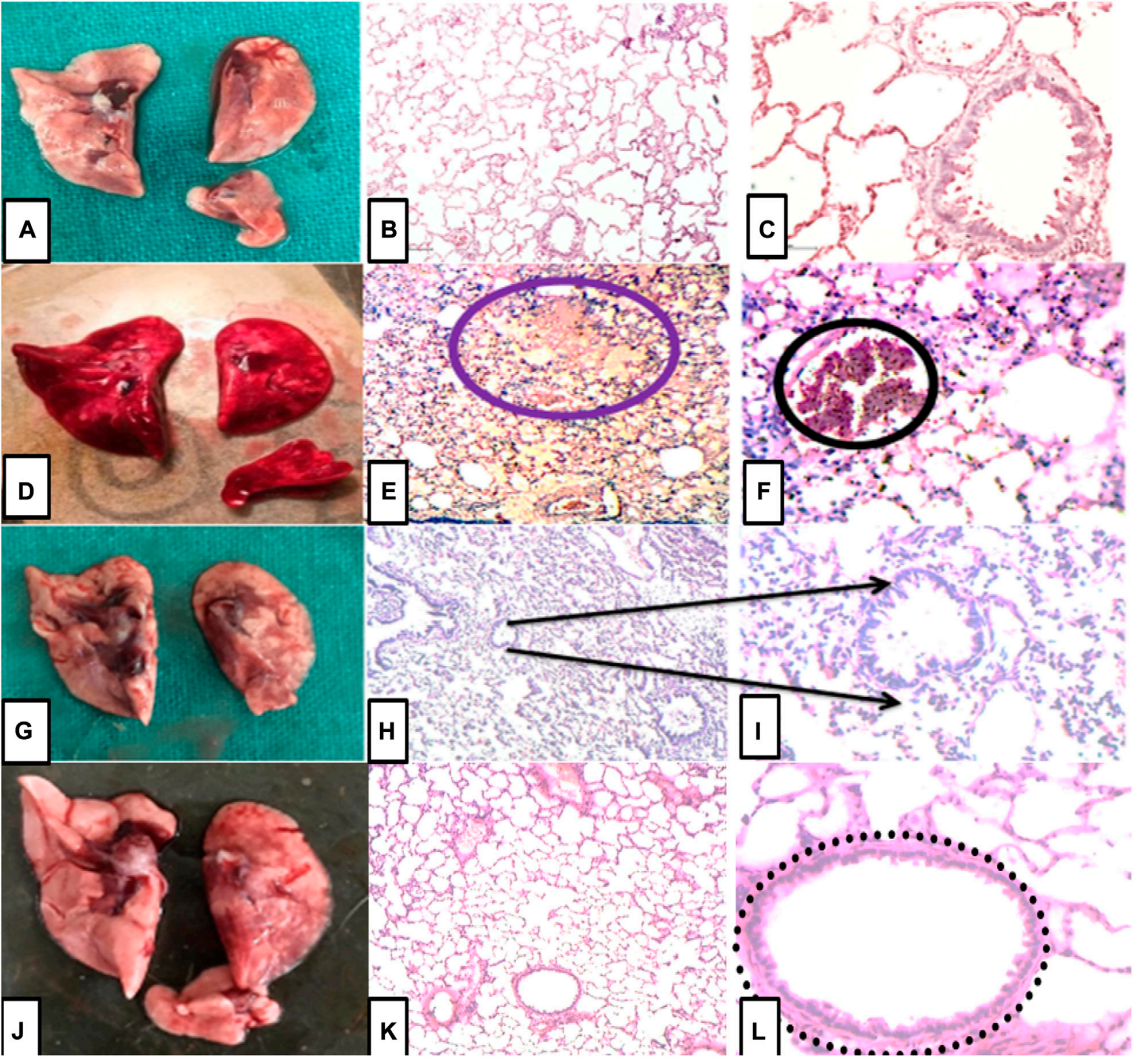
Pathological changes in the lungs of rats in each group. Surface morphology of lung in group I **(A)**, group II **(D)**, group III **(G)**, and group IV **(H)**. ×10 magnification of lung in group I **(B)**, group II **(E)**, group III **(H)** and group IV **(K)**. ×40 magnification of the lung in group I **(C)**, group II **(F)**, group III **(I)**, and group IV **(L)**. Here, the solid black circle denotes hemorrhagic clumping in the alveolar region, and the black arrows in **(I)** denote the damaged lining of the alveolus, whereas a dotted circle in **(J)** denotes the intact alveolus useful for gaseous exchange. The lung injury score (below) revealed significant relevance of group IV (B.T. 1.5 g/kg BW) to group I. Comparisons between groups were performed using one-way ANOVA (Turkey’s multiple comparison tests) to compare the mean values of each group with each other. (*p < 0.05, ***p < 0.01, and ****p < 0.001 is significantly different from group I; ^*###*^ *p < 0.01 and* ####*p < 0.001* are significantly different from group II).

In the normal control group (Figures 11A–C), the surface of the lung appeared to be pink and dry with intact structural intricacies. H & E staining in this group revealed an intact parenchyma, a thin and regular alveolar wall without neutrophil and inflammatory cell infiltration. No hyaline membrane was found in this group as well. Evident morphological and histopathological changes were observed in the oleic-acid treated group (Figures 11D–F). Lungs were hardened with hemorrhagic congestion, alveoli-capillary membranes were damaged, and hyaline membranes with blood clots were found in the alveoli, indicating the detrimental effect of oleic acid on the lungs. On the contrary, the dexamethasone-treated group (Figures 11G–I) and Bronco T (1.5 g/kg b. w.) treated group (Figures 11J–L) revealed a near-to-normal texture on morphological examination. Pretreatment significantly reduced inflammatory cell infiltrations and intact honeycomb parenchymal arrangement compared to the oleic acid treated group. B.T. pretreatment revealed intact lung endothelial and epithelial layers. Dexamethasone treated group showed damaged alveolar membrane denoted by black arrow with fewer infiltration as compared to oleic acid treated group. These observations were in harmony with semi-quantitative lung injury scoring, where oleic induces 67% and dexamethasone induces 62% score, whereas B.T. (1.5 g/kg b. w.) scored half the value around 33% suggesting it showed two-fold protection against the deteriorating effect of oleic acid.

### Effect of bronco T. On myeloperoxidase, malondialdehyde, and tumor necrosis factor

**FIGURE 12:**
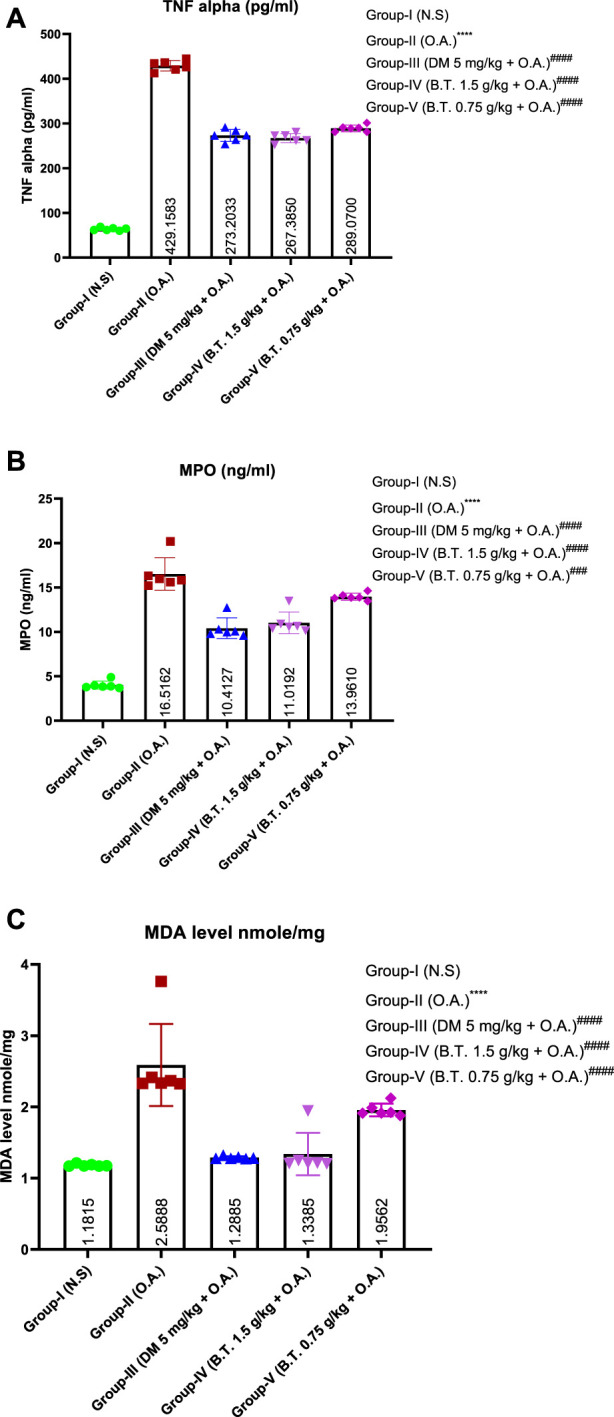
Effect of B.T. (1.5 g/kg B.W.) on **(A)** TNFα, **(B)** MPO and **(C)** MDA. Effect of B.T. (1.5 g/kg B.W.) on **(A)** TNFα, **(B)** MPO and **(C)** MDA. There was significant increase in these markers in oleic acid treated group which was reduced in group III and IV. Pretreatment with B.T. (1.5 g/kg B.W.) significantly reduced the level of inflammatory marker, neutrophil marker and oxidative stress marker in the lung homogenate. Comparisons between groups were performed using one-way ANOVA (Turkey’s multiple comparison tests) to compare the mean values of each group with each other. (^*^ *p < 0.05,* ^***^ *p < 0.01, and* ^****^ *p < 0.001* is significantly different from group I; ^*#*^ *,p < 0.05,* ^*###*^ *p < 0.01 and* ^*####*^ *p < 0.001* are significantly different from group II).

The level of neutrophil infiltration was measured by estimating the level of myeloperoxidase enzyme using an ELISA kit. The MPO level was elevated in group II; however, the group pretreated with BT (1.5 g/kg b. w.) pretreated group markedly (*p < 0.0001*) reduce the level of MPO compared to group II. A similar pattern was observed for dexamethasone. Increases in oxidative stress have been reported in ALI, so we estimate the concentration of MDA. It was significantly higher (MDA (*F*_*(4, 20)*_ *= 33.8, p < 0.0001*) in group II compared to group I. However, it was less formed in groups III and IV. TNFα levels increased significantly in the group treated with oleic acid that was attenuated in the pretreated group with BT (1.5 g/kg) and dexamethasone (5 mg/kg) as observed in Figure 12 **.**

### Effect of bronco T on oxidative stress in the lung of rats

**FIGURE 13:**
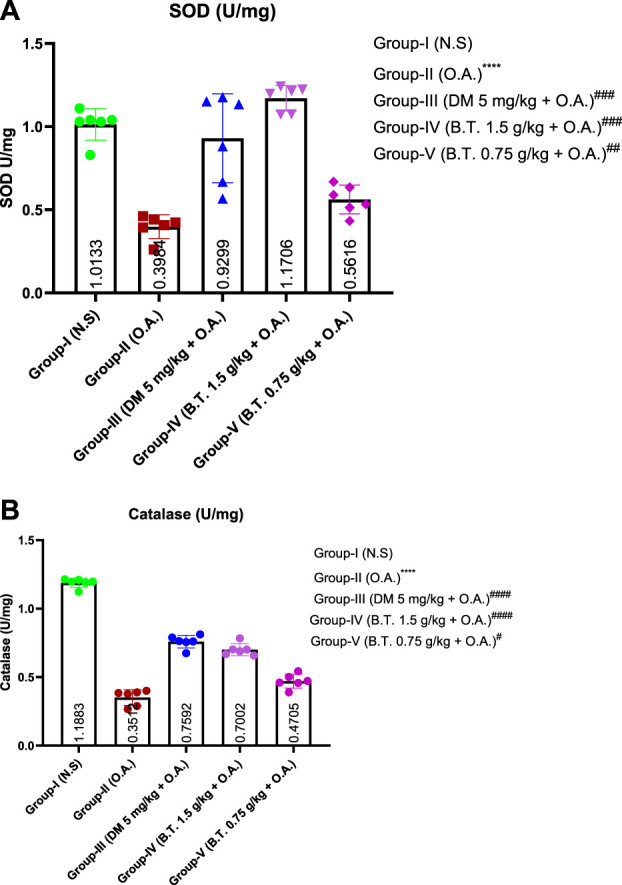
Effect of B.T. on antioxidant parameters. Effect of B.T. (1.5 g/kg B.W.) on antioxidant parameters **(A)** Superoxide dismutase (SOD) and **(B)** Catalase in BALF. The level of antioxidant enzymes was significantly reduced in oleic acid treated group. B.T. (1.5 g/kg B.W.) pretreated group significant restored the level of antioxidant enzymes. Comparisons between groups were performed using one-way ANOVA (Turkey’s multiple comparison tests) to compare the mean values of each group with each other. (^*^ *p < 0.05,* ^***^ *p < 0.01, and* ^****^ *p < 0.001* is significantly different from group I; ^*#*^ *,p < 0.05,* ^*###*^ *p < 0.01 and* ^*####*^ *p < 0.001* are significantly different from group II).

Oleic acid treatment produces oxidative stress in the lung that was prevented by pretreatment with BT (1.5 g/kg bw). The one-way ANOVA result showed a significant effect of B.T. pretreatment on SOD (*F*_*(4,20)*_) *=7.361, p < 0.0008*) and Catalase (*F*_*(4, 20)*_ *= 54.9, p < 0.0001*) levels. B.T. pretreatment increased the level of antioxidant enzymes superoxide dismutase and catalase Figure 13.

### Effect of bronco T on the level of COX-2 mRNA in the lung homogenate

**FIGURE 14:**
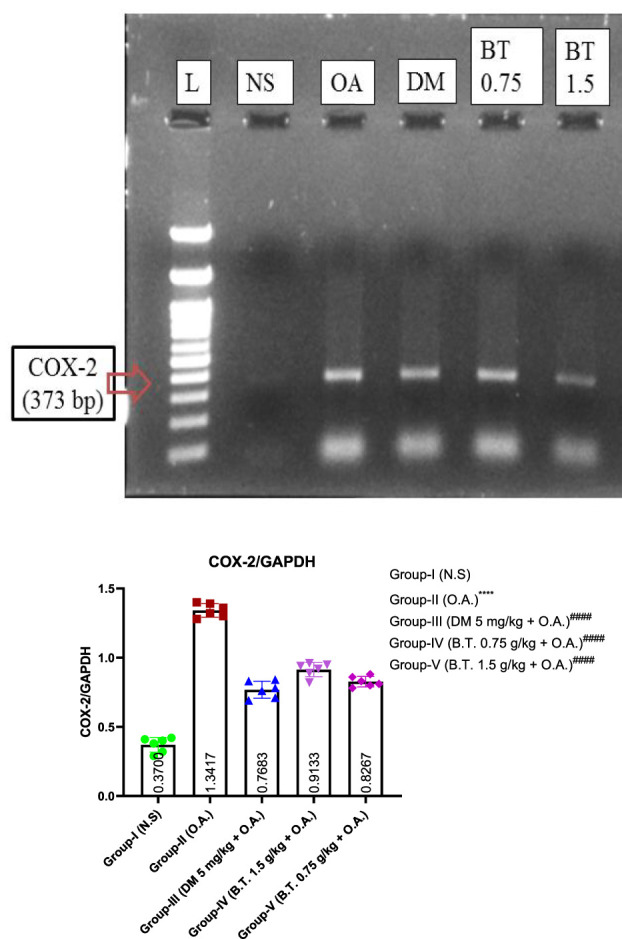
Effect of BT on the level of COX-2 mRNA. Effect of B.T. (1.5 g/kg B.W.) on the level of COX-2 mRNA in the lung tissue. The level of COX-2 was significantly increased in oleic acid treated group which was significantly reduced in B.T. (1.5 g/kg B.W.) pretreated group suggesting its protective action Comparisons between groups were performed using one-way ANOVA (Turkey’s multiple comparison tests) to compare the mean values of each group with each other. (^*^ *p < 0.05,* ^***^ *p < 0.01, and* ^****^ *p < 0.001* is significantly different from group I; ^*###*^ *p < 0.01 and* ^*####*^ *p < 0.001* are significantly different from group I).

Reverse transcriptase-polymerase chain reaction (RT-PCR) results indicate a considerable decrease in COX-2 mRNA expression in rats pretreated with dexamethasone group (III) compared to group (II) rats. A similar observation was found in BT (1.5 g/kg) pretreated group. Figure 14.

## Discussion

In the present work, the cardiorespiratory protective effect of the polyherbal formulation (Bronco T) was explored in the oleic acid-induced lung injury rat model. Oleic acid is a mono-saturated fatty acid that has been utilized to simulate acute lung injury as seen by fat embolism, which is clinically manifested as multiple trauma-induced respiratory distresses (T. Li et al., 2008).

Despite recent improvements in understanding the molecular pathophysiology of ALI, pharmaceutical treatments have yet to be developed. The majority of deaths in the COVID-19 pandemic were ascribed to ALI/ARDS (L. Li et al., 2020), which was caused by a lack of effective medicine and a poor prognosis. ALI/ARDS still has a 30–40% death rate worldwide, despite both classic and new treatment regimens (Ware and Matthay, 2005). As glucocorticosteroids are now the only reliable pharmaceutical intervention in the early stages of ALI (Janahi et al., 2018), the present study used dexamethasone as a positive control to compare the efficacy of BT in biophysical, biochemical, oxygenation index and histological changes related to ALI.

The Bronco T characterization study by HRLC-MS reported several phytoconstituents that have reported activity against respiratory tract disorders. Out of which seven compounds have been mentioned here. Among these beta-solamarine and Galangin-3- [galactosyl-(1–4)- rhamnoside showed significant binding energy with the reported targets in comparison to dexamethasone. Targets such as IL-6, TNFα, COX-2, and MPO were negatively regulated, while ENaC was positively regulated to reduce cytokine storm and increase alveolar fluid clearance, respectively. Suggesting that Bronco T is an effective option for the treatment of ALI according to *in silico* investigation. Previous research has found that high levels of neutrophil entrapment in the alveolar region are substantially linked to high mortality in ALI cases (Ma, 2001). In the present work, pretreatment with BT (1.5 g/kg b. w.) orally 3 hours before oleic acid reduced mortality, and rats survived for the entire 120 min observation period, implying a function in lowering the neutrophil level and its associated detrimental effect.

We evaluated cardiorespiratory parameters such as respiratory rate, mean arterial pressure, and heart rate in the animal model because the heart-lung interaction is difficult to evaluate during mechanical ventilation in patients with ARDS (Sipmann et al., 2018). These findings could lead to a more focused search for pharmacological interventions to treat ALI. We found that BT has a substantial effect on RR, MAP, and HR in this study, indicating that it has a cardio-respiratory protective action. Tachypnea is mentioned as a critical aspect in several publications citing the cause and clinical effects of ARDS (Saguil and Fargo, 2020), which arises as a compensatory mechanism in the early stages and leads to lung injury if left unchecked.

BT (1.5 g/kg) managed to overcome tachypnea and maintain a normal breathing rate during the current study experiment. Our results, which suggest that dexamethasone has a protective effect, are similar to those previously reported (Du et al., 2020), but there was early protection followed by tachypnea after 90 min, which eventually led to death.

In tissue perfusion and oxygenation, mean arterial pressure is crucial (Ryan et al., 2014). Pretreatment with BT (1.5 g/kg) significantly increased MAP after injury, which may play a role in the management of pulmonary hypotension and the prevention of cardiac function. Heart rate (HR) and PaO_2_/FiO_2_ can predict ICU mortality and a better prognosis in patients with ARDS, according to the Acute Physiological and Chronic Health Evaluation (APACHE III) scoring system (Chen et al., 2018). According to the results of this investigation, BT (1.5 g/kg) considerably reduced heart rate after injection of OA and improved oxygenation index in conjunction with dexamethasone treatment. Its potential clinical usage as an additional therapy in ICU patients is suggested. Oedema, increased permeability, alveolar infiltration of inflammatory cells, and higher levels of reactive oxygen species are all markers of lung injury (Borges et al., 2019). As a result, modifying these settings may help to alleviate ALI. BT (1.5 g/kg) substantially reduced pulmonary oedema in this trial by lowering the pulmonary water content as measured by the wet and dry ratio of the lung. It also revealed a considerable drop in superoxide and hydrogen peroxide levels.

ALI progresses as a result of neutrophil infiltration. When OA is given intravenously, it activates neutrophils, which produce many chemicals that damage lung tissue and cause persistent inflammation with fluid collection in the alveoli. The MPO level is a marker of neutrophilic infiltration at the inflammatory site. In the current investigation, BT (1.5 g/kg) reduced MPO to normal levels, preventing neutrophil accumulation and the formation of the hyaline membrane, as recently reported with continentalic acid (Ali et al., 2020).

According to numerous studies, there is an initial increase in the number of inflammatory cytokines in acute lung injury due to increased macrophage accumulation, particularly M1 (Mishra et al., 2021). Similar conclusions were found in the oleic acid group, which showed an increase in TNFα and COX-2 levels. However, pretreatment with dexamethasone reduced these cytokines, while BT (1.5 g/kg) similarly reduced inflammatory markers.

Pretreated rats with BT (1.5 g/kg) showed reduced hemorrhagic symptoms in both the alveoli and the extra alveoli regions, as shown by the low lung injury score and histological findings. When compared to the dexamethasone-treated group, the BT preparation (1.5 g/kg) showed significant protection against oleic acid-induced histological changes in lung tissue, which was similar to an earlier report (Kajaria et al., 2014) citing intact alveolar-capillary membrane, no hemorrhagic manifestation, absence of protein-rich fluid in the interstitium, and fewer neutrophil infiltrations.

The 1.5 g/kg dose of B.T., which was superior to the 0.75 g/kg dose, produced the best results in the dose-response trial with BT pretreatment. For the first time, our research shows that BT (1.5 g/kg) protects against the cardiorespiratory consequences induced by oleic acid of ALI/ARDS.

## Conclusion

Bronco T (1.5 g/kg b. w.) prevented mortality and help to recover from oleic acid-induced deleterious cardiorespiratory effect, and inflammatory and oxidative stress in rat lungs. Hence it could be used as add on a treatment option for managing acute lung injury under clinical supervision. Further research is required to identify the active phytoconstituents and their mechanism of action for a complete understanding of the molecular mechanisms involved.

## Data Availability

The original contributions presented in the study are included in the article/Supplementary Material, further inquiries can be directed to the corresponding author.

## Abbreviations

ALI, Acute Lung Injury; ARDS, Acute Respiratory Distress Syndrome; BT, Bronco T; BW, Body Weight; COX-2, Cyclooxygenase 2; HR, Heart Rate; HR LCMS, High-Resolution Liquid Chromatography-Mass Spectroscopy; MAP, Mean Arterial Pressure; MDA, Malondialdehyde; MPO, Myeloperoxidase; PaO2/FiO2, Arterial Oxygen Partial Pressure/Fractional Inspired Oxygen Partial Pressure; RR, Respiration Rate; SpO2, Oxygen Saturation; TNFα, Tumor Necrosis Factor Alpha.
